# Synergistic antitumor activity by combining trastuzumab with retinoic acid in HER2 positive human breast cancer cells

**DOI:** 10.18632/oncotarget.25480

**Published:** 2018-05-29

**Authors:** Fiorella Vanderhoeven, Analía Lourdes Redondo, Ana Laura Martinez, Laura María Vargas-Roig, Angel Matias Sanchez, Marina Inés Flamini

**Affiliations:** ^1^ Instituto de Medicina y Biología Experimental de Cuyo, Centro Científico Tecnológico, Mendoza, Argentina; ^2^ Facultad de Ciencias Médicas, Universidad Nacional de Cuyo, Mendoza, Argentina

**Keywords:** anti-ErbB2 therapies, Moesin, focal adhesion kinase, adhesion, migration

## Abstract

Breast cancer can be classified into molecular subtypes. Tumors overexpressing HER2 protein are more aggressive and metastatic; hence, patients have a poor prognosis. Anti-HER2 strategies, such as the monoclonal antibody Trastuzumab (Tz), have therefore been developed. Despite this progress, not all patients respond to the treatment. Retinoic acid (RA) has been proposed as an adjuvant treatment of breast carcinoma because of its ability to inhibit cell growth. We evaluated the effect of Tz in combination with RA on the viability, adhesion, migration, invasion and expression of migration-related proteins in SKBR3 and BT-474 human breast cancer cells. MTT, pharmacological interaction analysis, immunofluorescence, adhesion/migration/invasion and Western blot assays were performed. The coadministration of both drugs synergistically decreased cell survival. Tz+RA significantly decreased adhesion/migration/invasion in both cell types. Tz+RA strongly reduced FAK and HER2 expression and induced nuclear FAK translocation. In addition, a granular distribution of HER2 receptor was observed after the combined treatment. In conclusion, the coadministration of both drugs in patients with this type of cancer could contribute to the improvement of their prognosis and reduce the adverse effects of therapy because the applied Tz doses would be lower due to the adjuvant effect of RA.

## INTRODUCTION

Breast cancer is one of the most common cancers in women worldwide [1]. It is a heterogeneous disease that is classified into molecular subtypes according to the presence or absence of estrogen receptors (ER), progesterone receptors or human epidermal growth factor receptor 2 (HER2, also known as ErbB-2). HER2 protein is a key molecule in breast cancer because signaling through this receptor contributes to oncogenic transformation [2].

Amplification of *HER2* gene occurs in 25–30% of breast cancers and results in high levels of HER2 protein expression [3]. This is accompanied by an increase in HER2 signaling and promotion of malignant cell growth and survival [4]. Patients whose tumors are characterized by *HER2* gene amplification and protein overexpression therefore develop a more aggressive type of cancer, which is associated with poor prognosis [5]. HER2 is an attractive target for immunotherapy because it is expressed at relatively low levels in normal tissues. One of the existing anti-HER2 strategies is the use of the monoclonal antibody Trastuzumab (Tz) or Herceptin^®^, which binds to the extracellular domain of HER2. Tz is the first line of treatment for HER2-positive breast cancers. It improves overall survival when used as a single agent [6] or in combination with chemotherapy [7, 8]. Despite its success, 40-60% of patients do not respond to the treatment or develop resistance to it [7, 9]. This fact calls for new therapeutic approaches based on the combination of different drugs and the combination of targeted therapies have great potential.

Retinoids, mainly retinoic acid (RA), have been proposed as an adjuvant treatment of breast carcinoma because of their ability to inhibit cell growth and induce morphological or phenotypic differentiation [10]. RA, a pleiotropic signaling molecule, regulates critical genetic programs that control development, homeostasis, proliferation, differentiation, cell death and/or survival [11, 12]. Its antitumor activity is primarily mediated by retinoic acid receptors (RAR), which belong to the nuclear receptor superfamily RARα, RARβ and RARγ. RARs act as ligand-inducible transcriptional regulators and heterodimerize with retinoid X receptors (RXRs). As such, they regulate the expression of a subset of target genes [13].

An effective clinical use of retinoids in breast carcinoma treatment requires the identification of subpopulations of patients who might be sensitive to therapy and therefore would benefit from it. Preclinical and clinical data indicate that high levels of RARα in the tumor predict sensitivity to the treatment with retinoids [14]. A significant fraction of HER2-positive breast carcinomas is characterized by co-amplification of the *RARα* gene, which leads to increased expression of the RARα protein and is associated with sensitivity to the antiproliferative action of RA [15]. This is of particular relevance in the context of ER-negative tumors, which are refractory to hormonal therapies. In ER-/HER2+/RARα+ tumors, the sensitivity to anti-HER2+ therapies is even greater when RA is administered simultaneously [15].

Retinoids have been implicated in the inhibition of cell adhesion and migration. For instance, RA and other biologically active retinoids administered over prolonged periods inhibit migration in human colon carcinoma cells [16] as well as in MCF7 and MDA-MB-231 human breast cancer cells [16–18]. Because relapse and patient mortality result in part from tumor spread and metastasis, it is fundamental to study the effect of Tz and RA in adhesion, migration and invasion of human breast cancer cells.

Moesin is an important protein in the process of tumor spread, invasion and metastasis. It induces actin depolymerization, and its translocation towards the edge of the cell membrane and is responsible for the formation of cortical actin complexes [19]. Another key protein is focal adhesion kinase (FAK), which participates in the assembly and disassembly of focal adhesion complexes, reorganizing them in the migration direction. Its overexpression is correlated with more aggressive tumors [20].

Our group has recently shown that RA inhibits cell migration by remodeling the actin cytoskeleton and regulating expression of Moesin and c-Src/FAK in human breast cancer cells T-47D and MCF7 [21, 22]. Based on our current and previous findings, we hypothesized that the combination of Tz and RA would have a synergistic effect in decreasing the viability, and reducing the adhesion and migration of HER2+/RAR+ human breast cancer cells by modifying the expression/localization of proteins related to the cellular movement.

## RESULTS

### Tz, RA and the combination of both drugs decrease SKBR3 and BT-474 cell viability

**Figure 1:**
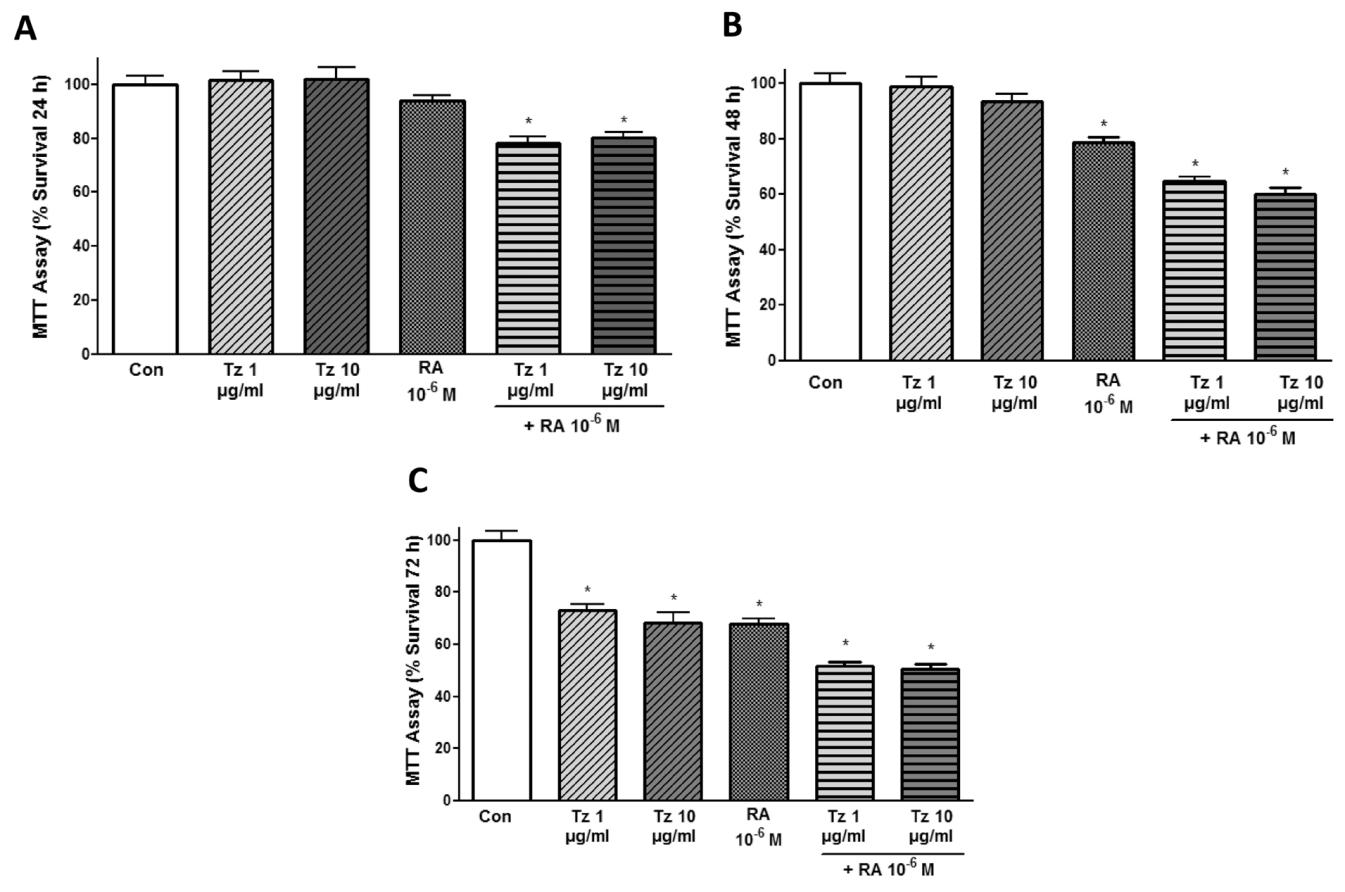
Tz and RA inhibit SKBR3 cell viability MTT assay in SKBR3 cells at different times. SKBR3 cells were plated at densities of 20,000 cells/well and treated with 1 or 10 μg/ml trastuzumab (Tz), 10^-6^M retinoic acid (RA) or the combination of Tz+RA for **(A)** 24 h **(B)** 48 h and **(C)** 72 h. Absorbance was measured at 540 nm. The results express the percentage (%) of surviving cells in relation to the control (Con, 100% survival). All data shown are representative of three independent experiments. Error bars indicate standard deviations. ^*^P <0.05 vs. Control.

Our first objective was to establish the adequate treatment period to evaluate cell survival in breast cancer cells. We treated SKBR3 cells with different concentrations of Tz 1-10 μg/ml, 10^-6^M RA and the combination of both drugs during 24, 48 or 72 h. Cell survival was significantly reduced after treatment for 24 h only with combined treatments of 1-10 μg/ml Tz + 10^-6^M RA (Figure 1A). Treatment with RA alone as well as the combination of both drugs significantly inhibited cell survival after 48 h (Figure 1B). All conditions significantly reduced cell viability when the treatment was applied for 72 h, and a 50% reduction in cell viability was achieved with the combination of both drugs (Figure 1C).

**Figure 2:**
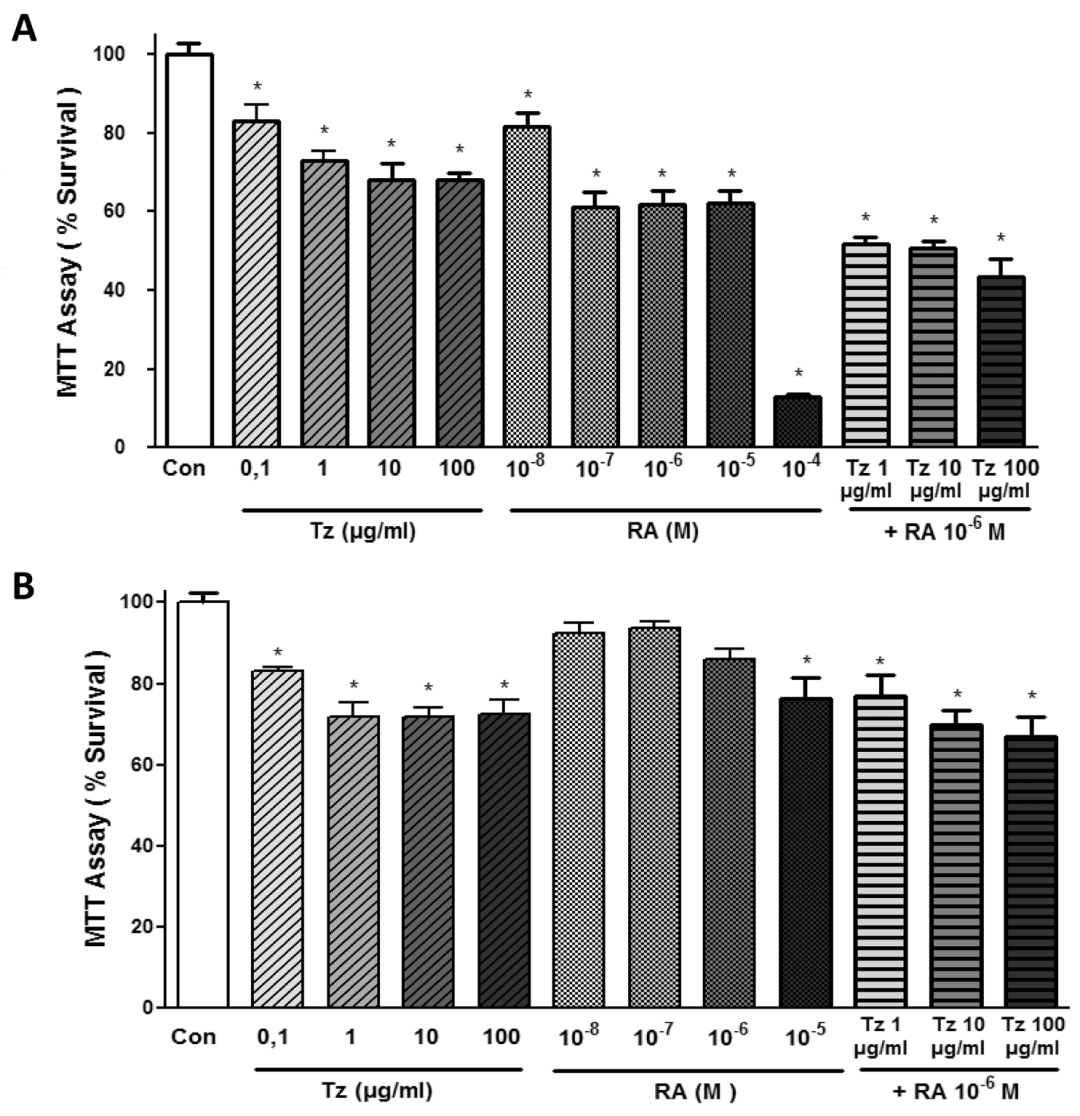
Tz, RA and the combination of both drugs decrease SKBR3 and BT-474 cell viability MTT assay at different doses. **(A)** SKBR3 cells and **(B)** BT-474 cells were treated with increasing doses of Tz (0.1-1-10-100 μg/ml), RA (10^-8^-10^-7^-10^-6^-10^-5^-10^-4^ M) or the combination of Tz (1-10-100 μg/ml) with 10^-6^M RA for 72 h. The results express the percentage (%) of surviving cells compared to the control (Con, 100% survival). All data shown are representative of three independent experiments. Error bars indicate standard deviations. ^*^P <0.05 vs. Control.

We then treated SKBR3 and BT-474 cells for 72 h with increasing doses of Tz (0.1-1-10-100 μg/ml), RA (10^-8^-10^-7^-10^-6^-10^-5^-10^-4^ M) or the combination of 1, 10 or 100 μg/ml Tz with 10^-6^M RA. In SKBR3 cells, all tested Tz concentrations and RA treatments significantly reduced cell viability (Figure 2A). The combination of 10^-6^M RA with different doses of Tz (1-10-100 μg/ml) significantly reduced cell viability between 50 and 58% (Figure 2A). In BT-474 cells, we observed a significant reduction in cell survival with all Tz treatments (Figure 2B). However, only the treatment with 10^-5^M RA, but not other doses of this drug applied individually, significantly reduced cell viability. All combinations of 10^-6^M RA with different doses of Tz significantly reduced cell viability in BT-474 cells (Figure 2B).

### Tz and RA exert a synergistic inhibition of cell viability

**Figure 3:**
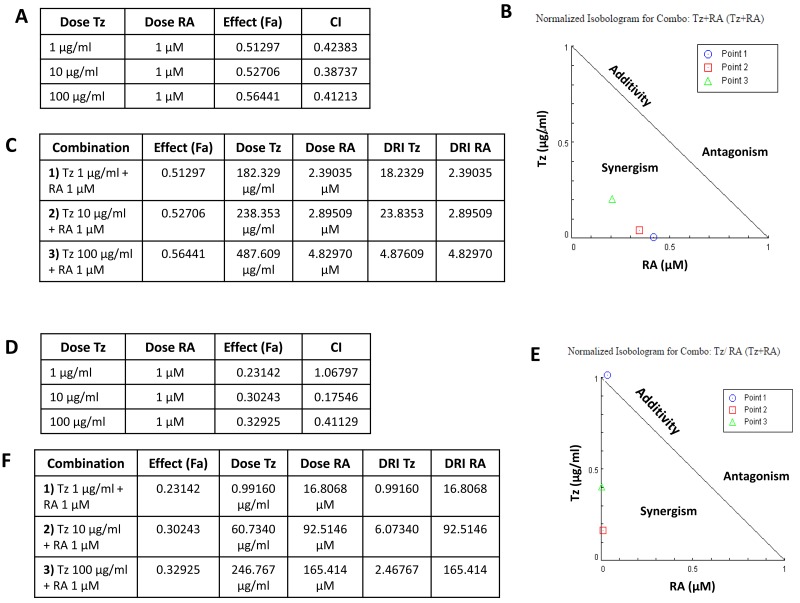
Tz and RA exert a synergistic inhibition of cell viability Analysis of the pharmacological interaction between Tz and RA. The combination index (CI) was calculated with the CompuSyn program using the 72 h-treatments of the MTT assay. When two drugs are simultaneously delivered, CI>1 indicates antagonism in the combination; CI = 0.9-1.1 additivity; and CI<1 synergism. **(A-C)** SKBR3 cells, **(D-F)** BT474 cells. (A, D) The tables show the combinations of doses tested, the effect produced by each combination, affected fraction (Fa), and the corresponding CI. (B, E) Isobolograms for the Tz and RA combination. Point 1: 1 μg/ml Tz + 10^-6^M RA; point 2: 10 μg/ml Tz + 10^-6^M RA; point 3: 100 μg/ml Tz + 10^-6^M RA. (C, F) Tables with the dose reduction index (DRI) for each combination.

The combination index (CI) obtained through the CompuSyn software was used to examine the degree of drug interaction when treating cells with both drugs simultaneously. In SKBR3 cells, treatments with 10^-6^M RA +1-10 or 100 μg/ml Tz all reached CI values <1 (Figure 3A), suggesting that the combination of both drugs synergistically decreases cell viability. This implies that the interaction between the two drugs produced a greater effect than each one of the drugs separately, as shown in the corresponding isobologram (Figure 3B).

One of the main goals of having synergistic drugs is to reduce the dose used and thus to decrease toxicity while maintaining efficacy. In this context, the dose reduction index (DRI) is important from a clinical point of view. It indicates how much the dose of a drug could be reduced in a scenario of a synergistic combination while producing a similar effect, compared to the dose used for each drug separately. In SKBR3 cells, the fraction affected by the combinations of 1-10 or 100 μg/ml Tz + 10^-6^M RA were 0.512, 0.527 and 0.564, respectively (Figure 3C). The analysis of the DRI of the 1-10 μg/ml Tz + 10^-6^M RA combination reveals that the dose of Tz can be 10 times lower when the drug is used within this synergistic combination (18.2329-23.8353 μg/ml), compared to the application of Tz alone (182.329-238.353 μg/ml) while still achieving the same effect. The DRI of 100 μg/ml Tz + 10^-6^M RA indicates that the effect is 100 times more potent (4.87609 μg/ml) within this synergic scenario than when Tz is applied alone (487.609 μg/ml). Hence, the dose of Tz could be decreased 100 times and achieving the same effect (Figure 3C).

In BT-474 cells, the combination of 10-100 μg/ml Tz with 10^-6^M RA exerts a strongly synergistic effect (CI<1) on reducing cell viability, whereas the effect of the treatment at lower doses (1 μg/ml Tz + 10^-6^M RA) is additive (CI =1.06797) (Figure 3D–3E). In BT-474 cells, the fraction affected by the combinations of 1-10 or 100 μg/ml Tz + 10^-6^M RA were 0.231, 0.302 and 0.329, respectively (Figure 3F). In the combination 1 μg/ml Tz + 10^-6^M RA the individual doses and the DRI calculated were identical, indicating that the effect produced by the combined treatment is equal to adding the doses of the individual treatments. This demonstrates the additive effect of this combination, a result that agrees with the CI obtained (CI= 1). The DRI of the combinations of 10 or 100 μg/ml Tz + 10^-6^M RA indicate that the dose of Tz can be reduced 10-fold or 100-fold, respectively, when used within this synergistic combination compared to its individual application (DRI =6.07340, 2.46767 and 60.7340, 246.767 respectively) (Figure 3F).

### Tz, RA and the combination of both drugs decrease adhesion, migration and invasion of breast cancer cells

**Figure 4:**
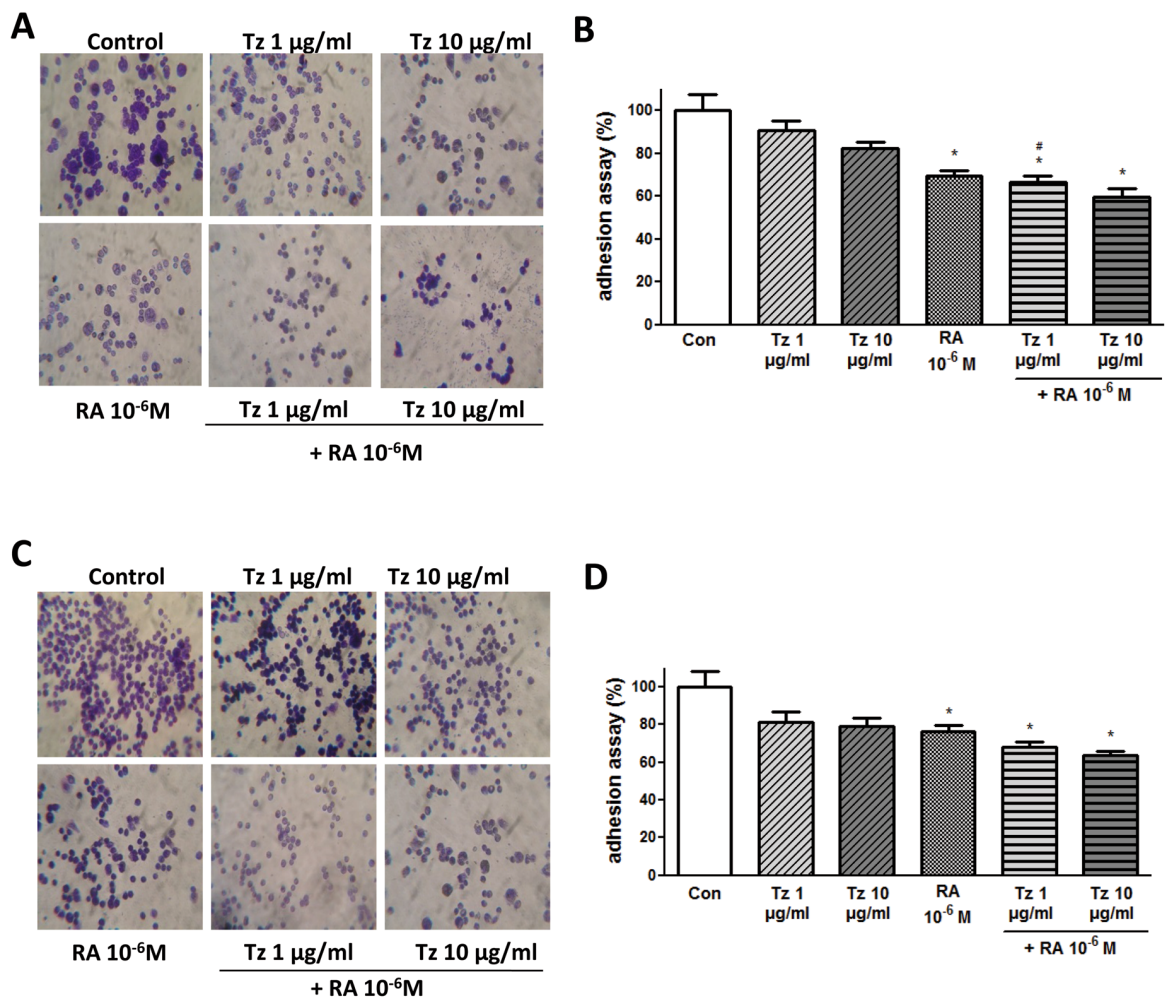
RA and the combination of both drugs inhibit cell adhesion **(A, B)** SKBR3 and **(C, D)** BT-474 cells were treated for 72 h with 1-10 μg/ml Tz, 10^-6^M RA or the combination of both drugs. After the treatment, cells were placed on coverslips previously covered with gelatin and a cell adhesion assay was performed. (A, C) Representative images of the adhered cells. (B, D) Percentage of attached cells (absorbance at 570 nm). Experiments were performed in triplicate. ^*^P <0.05 vs. Control (Con). ^#^P <0.05 vs. 1 μg/ml Tz.

To assess the relevance of Tz and RA on breast cancer cell adhesion, migration and invasion we treated SKBR3 and BT-474 cells with different concentrations of Tz, RA or both for 72 h, and then allowed to adhere for 1 h to an extracellular matrix-like surface. In SKBR3 cells, treatment with 10^-6^M RA significantly inhibited adhesion compared to control cells by 30%. The combination of 1-10 μg/ml Tz + 10^-6^M RA did so by 34% and 40%, respectively. Although 1 μg/ml Tz did not significantly reduce adhesion, the addition of RA produced a significant inhibition, suggesting that the mere fact of adding RA improves the effect of Tz (Figure 4A–4B). In BT-474 cells, treatment with 10^-6^M RA significantly inhibited adhesion by 24%, whereas the combination of 1-10 μg/ml Tz + 10^-6^M RA induced a significant inhibition of 32-36% respectively (Figure 4C–4D).

**Figure 5:**
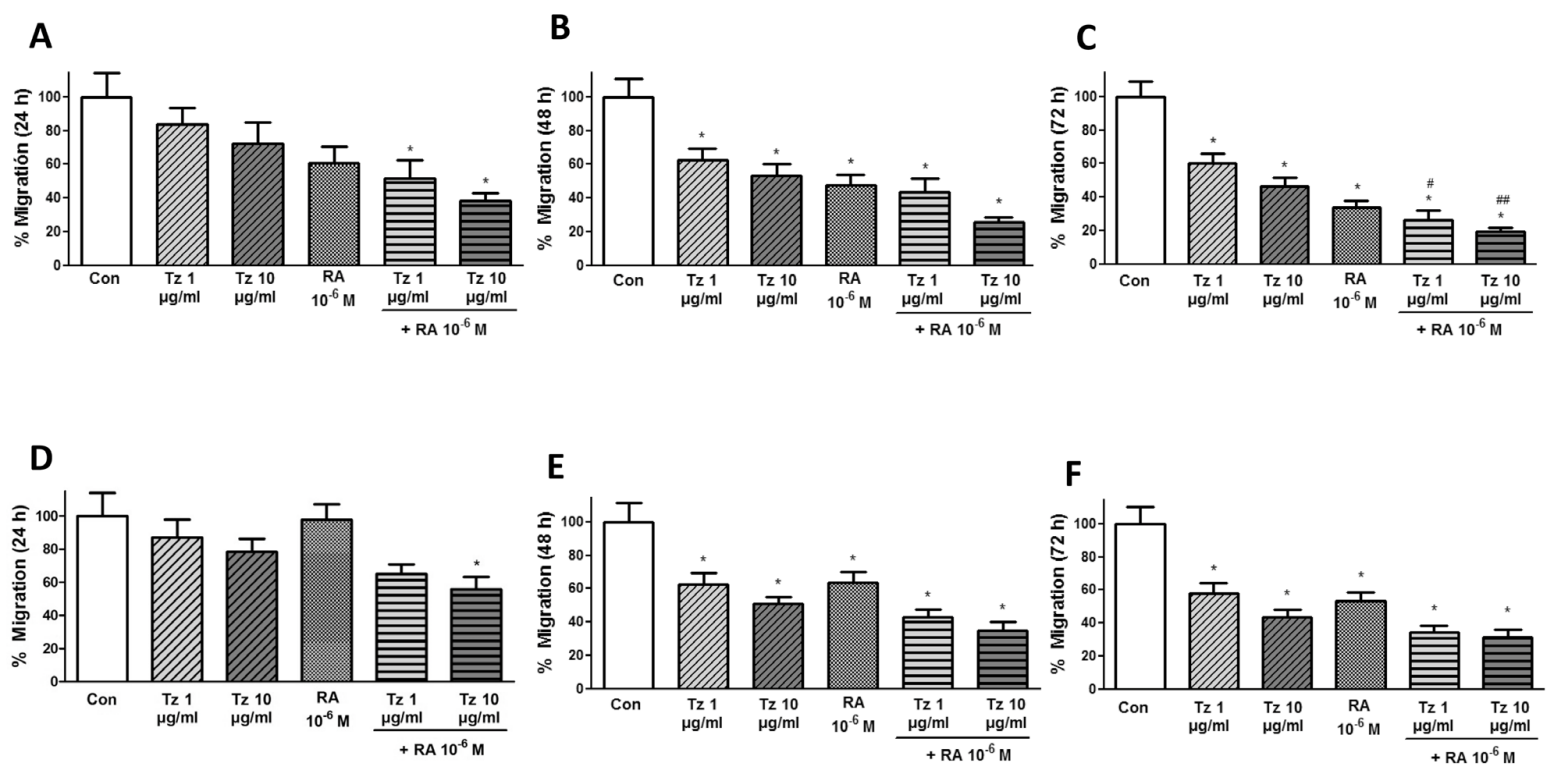
Tz and RA reduce cell migration **(A-C)** SKBR3 and **(D-F)** BT-474 cells were grown in 96-well plates until they formed a monolayer; then, a “wound” was made. Treatments were applied for 72 h and cell migration was monitored by taking photographs. (A, D) 24 h (B, E) 48 h (C, F) 72 h of treatment. Gap closure was quantified with the use of NIH image J software. Experiments were performed in triplicate. ^*^P <0.05 vs. Control (Con). ^#^P <0.05 vs. 1 μg/ml Tz. ^##^P <0.05 vs. 10 μg/ml Tz.

The wound healing assay was performed for 24-72 h to assess migration. Both cell lines were treated with 1-10 μg/ml Tz, 10^-6^M RA or the combination of both drugs. In additional experiments (see Supplementary Data), we treated the cells with/without FAK inhibitor (FAKi) or specific siRNA for FAK due to its intervention in focal adhesion formation. The cell migration distance was analyzed after 0, 24, 48 and 72 h. In SKBR3 cells, only the drug combination induced a significant decrease in migration after 24 h of treatment (Figure 5A). Migration was significantly decreased in all conditions after 48 and 72 h of treatment compared to control cells, and cell migration was reduced by 80% with the combined treatment. It is important to note that the addition of RA to Tz produced a significant inhibition of migration compared to 1-10 μg/ml Tz alone, reaffirming that RA improves Tz effects (Figure 5B–5C). In BT-474 cells, the 24 h treatment significantly reduced cell migration only when 10 μg/ml Tz was combined with 10^-6^M RA (Figure 5D). Migration significantly decreased in all conditions after 24 and 72 h of treatment, and a reduction in cell migration of nearly 70% was achieved with the combined treatment (Figure 5E–5F).

We performed siRNA assays or used specific inhibitors for FAK protein to verify whether RA or Tz decrease adhesion and migration through FAK. When SKBR3 and BT-474 cells were treated with FAKi and/or a specific siRNA against FAK, in combination or not with Tz and RA, migration was either inhibited or reduced, suggesting that FAK is not fundamental or at least it is not the only protein by which Tz and RA induce the inhibition of cell migration (Supplementary Figures 1-2).

**Figure 6:**
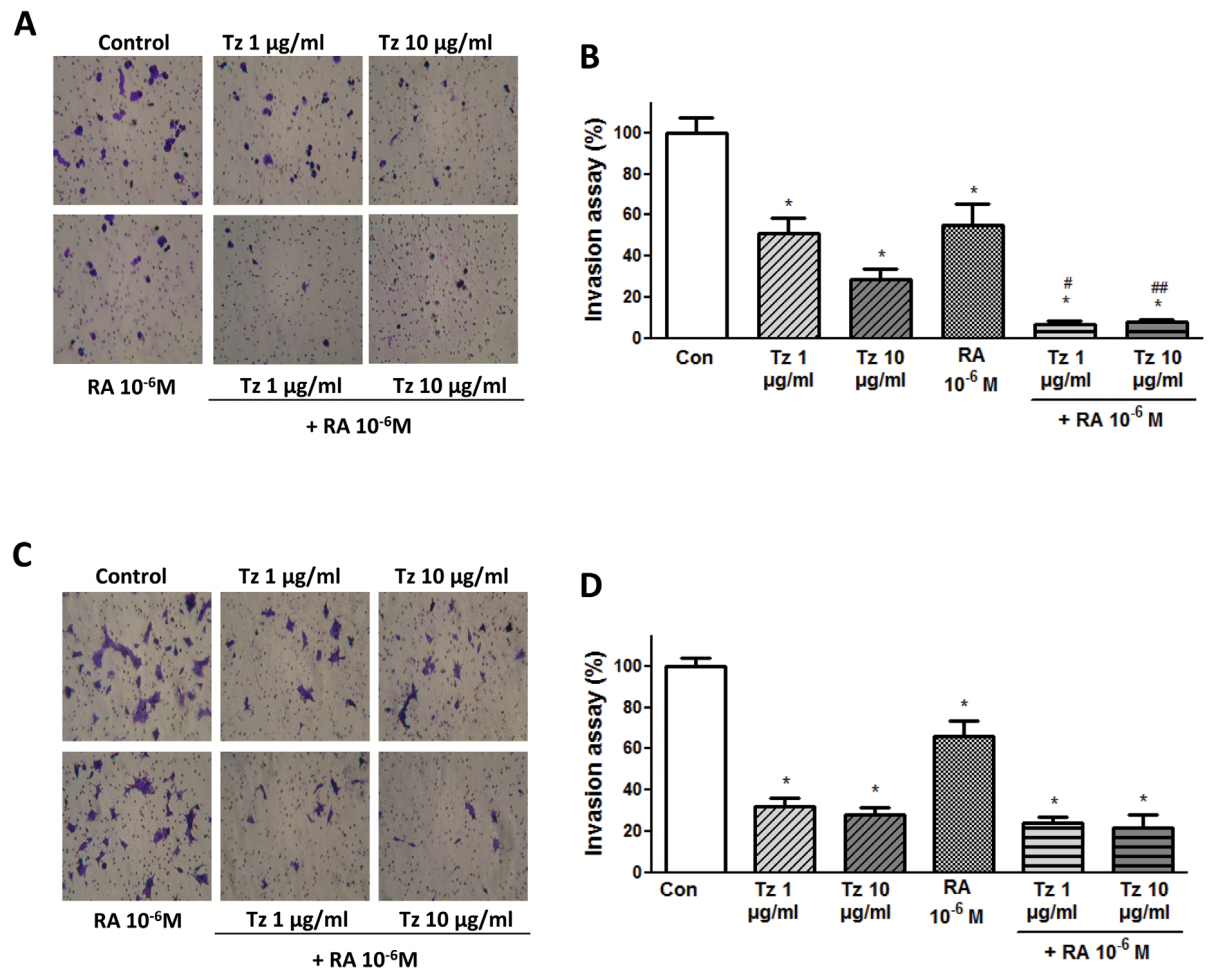
Tz and RA inhibit cell invasion **(A, B)** SKBR3 and **(C, D)** BT-474 cells were treated for 72 h with 1 or 10 μg/ml Tz, 10^-6^M RA or the combination of both drugs. After the treatment, cells were seeded on top of a Matrigel invasion chamber and a cell invasion assay was performed. (A, C) Representative images of invasion in the different conditions. (B, D) Percentage of invading cells observed, photographed under the microscope at 100X magnification and counted in the central field. Experiments were performed in triplicate. ^*^P <0.05 vs. Control (Con). ^#^P <0.05 vs. 1 μg/ml Tz. ^##^P <0.05 vs. 10 μg/ml Tz.

Finally, we performed a cell invasion assay. In SKBR3 cells, all conditions significantly reduced cell invasion compared to control cells, and the combined treatment reduced it by 93%. We highlight that the addition of RA to Tz produced a significant inhibition of invasion compared to 1-10 μg/ml Tz alone (Figure 6A-6B). In BT-474 cells, cell invasion was also significantly reduced by all treatments, and the combination of 1-10 μg/ml Tz + 10^-6^M RA induced a significant inhibition of 76% and 79%, respectively (Figure 6C–6D).

### Tz and RA induce nuclear FAK translocation in SKBR3 and BT-474 cells

**Figure 7:**
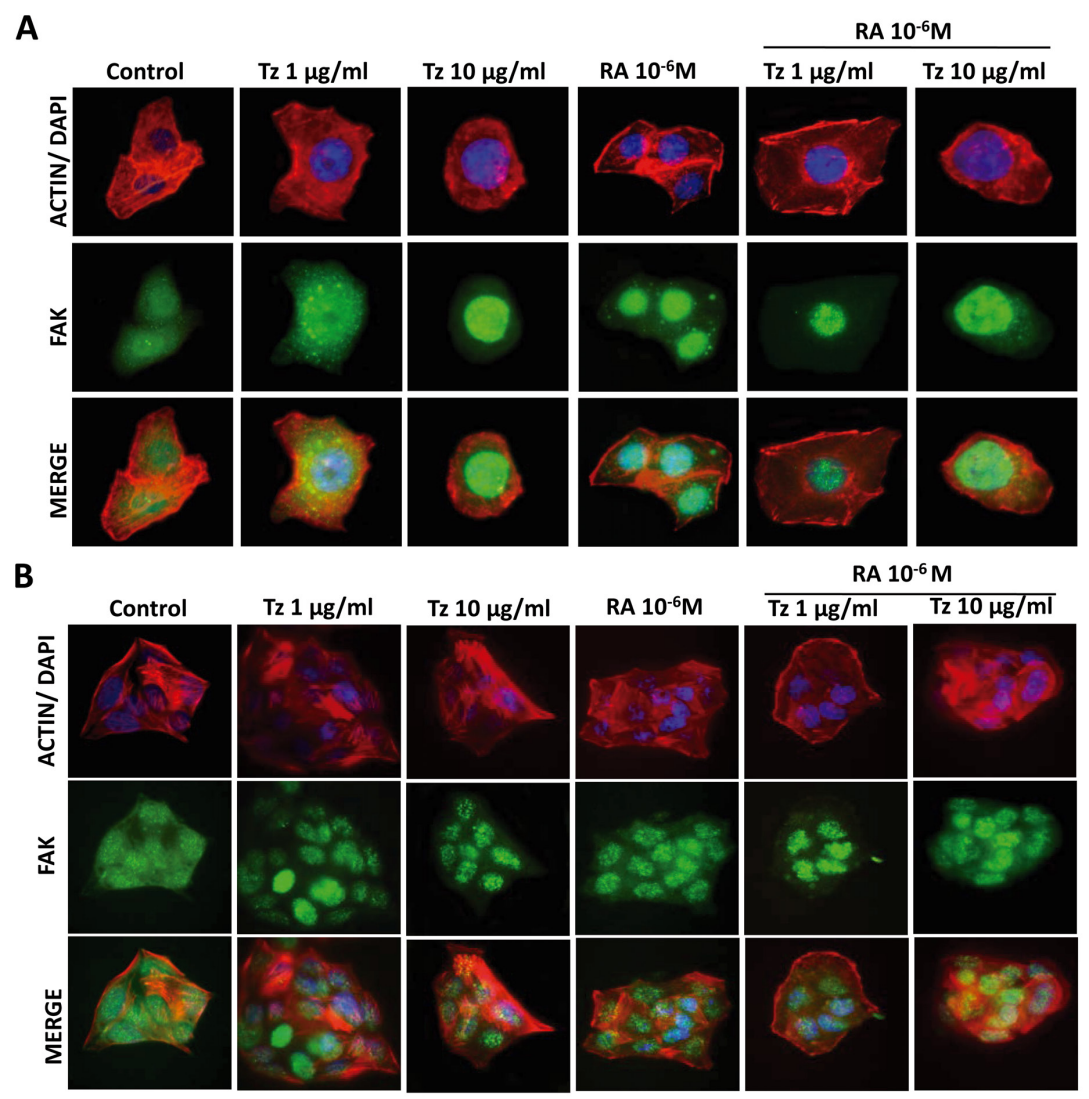
Tz and RA induce FAK nuclear localization **(A)** SKBR3 and **(B)** BT-474 cells were treated for 72 h with 1 or 10 μg/ml Tz, 10^-6^M RA or the combination of both drugs. The cells were stained with FAK linked to Alexa Fluor 488 (green); Actin was stained with Texas Red Phalloidin (red); and nuclei were counterstained with DAPI (blue). The images were captured using the Nikon Eclipse E200 fluorescence microscope coupled to a high resolution digital camera. All experiments were repeated three times with consistent results. Representative images are shown.

SKBR3 and BT-474 cells were treated for 72 h with 1-10 μg/ml Tz, 10^-6^M RA or the combination of both drugs. We did not observe changes in the structure of the actinic cytoskeleton after administration of 1-10 μg/ml Tz, 10^-6^M RA or both drugs, neither in SKBR3 nor in BT-474 cells. FAK was homogeneously distributed throughout the cell cytoplasm in control samples of both cell lines. In SKBR3 cells, the same distribution was observed after treatment with 1 μg/ml Tz; however, 10 μg/ml Tz, 10^-6^M RA and the combination of both drugs induced a translocation of FAK from the cytoplasm to the nucleus (Figure 7A). In BT-474 cells, all treatments induced nuclear FAK translocation, although the percentage of cells showing this distribution was lower than in SKBR3 cells (Figure 7B).

### Combined treatment with Tz and RA induces HER2 receptor redistribution in SKBR3 and BT-474 cells

**Figure 8:**
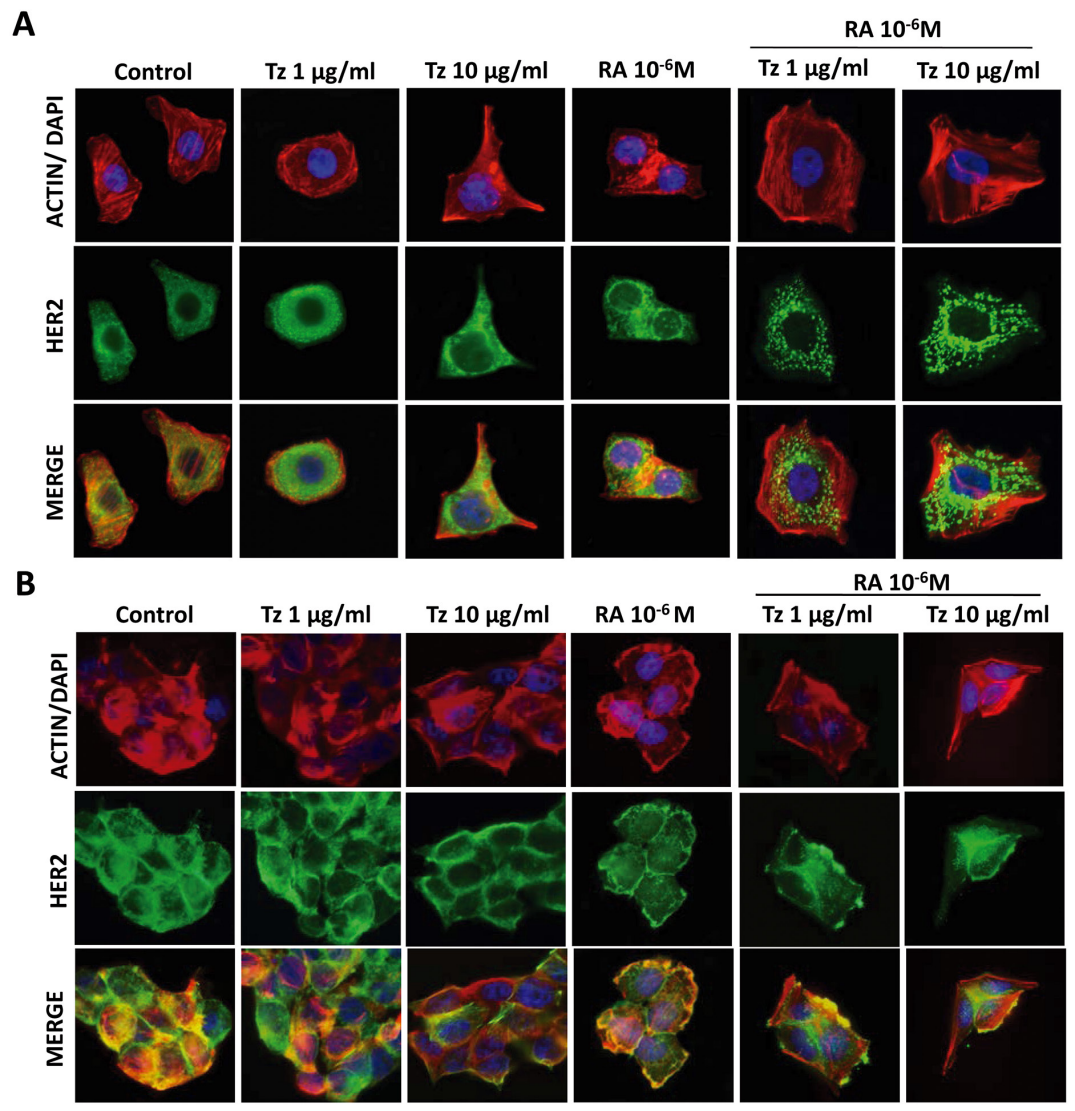
Tz and RA induce HER2 granular distribution **(A)** SKBR3 and **(B)** BT-474 cells were treated for 72 h with 1 or 10 μg/ml Tz, 10^-6^M RA or the combination of both drugs. The cells were stained with HER2 linked to Alexa Fluor 488 (green); Actin was stained with Texas Red Phalloidin (red); and nuclei were counterstained with DAPI (blue). The images were captured using the Nikon Eclipse E200 fluorescence microscope coupled to a high resolution digital camera. All experiments were repeated three times with consistent results. Representative images are shown.

**Figure 9:**
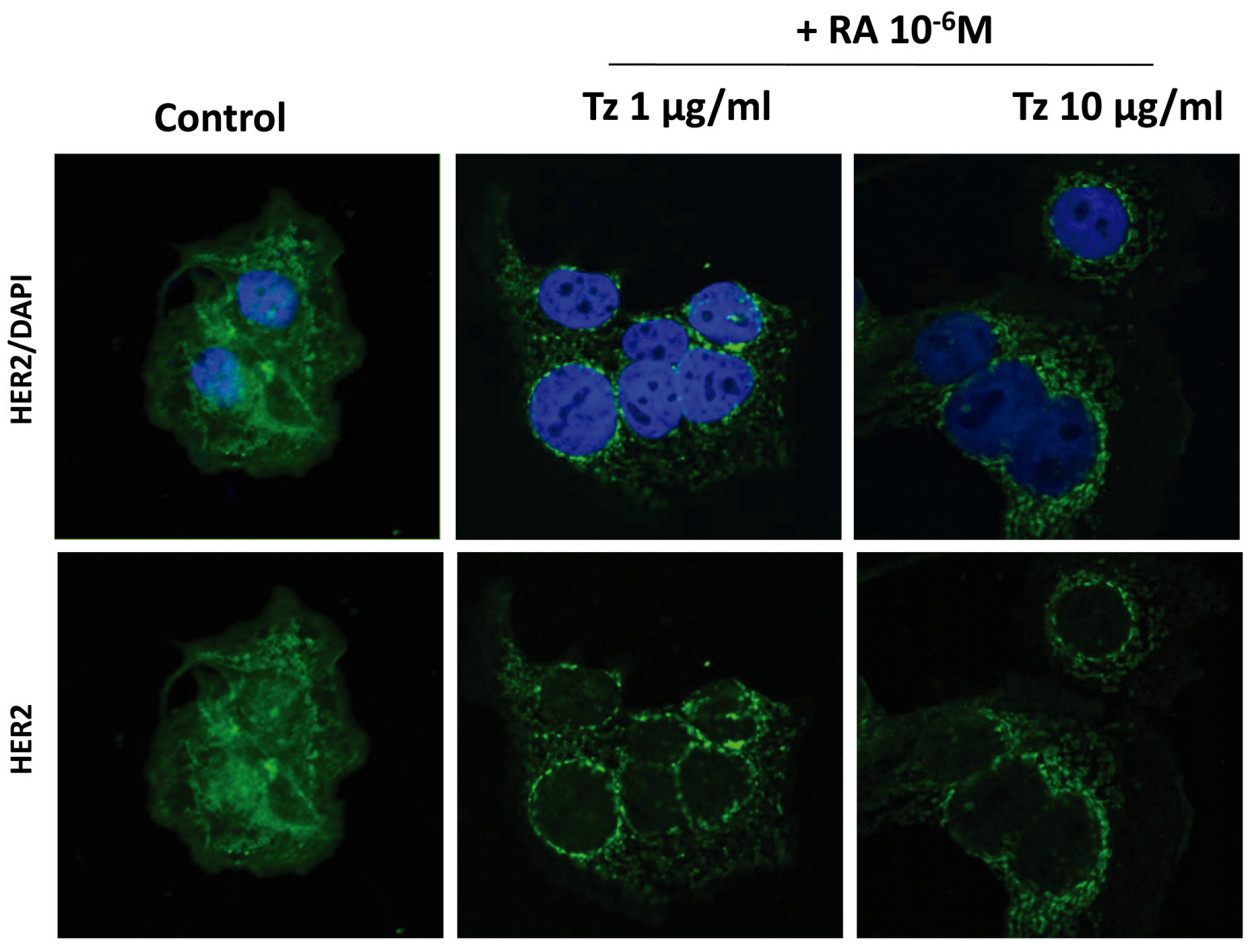
Tz and RA induce HER2 internalization SKBR3 cells were treated for 72 h with 1 or 10 μg/ml Tz combined with 10^-6^M RA. The cells were stained with HER2 linked to Alexa Fluor 488 (green) and nuclei were counterstained with DAPI (blue). The images were examined under fluorescence microscopy (FV1000 Olympus Confocal Microscope) and the FV 10-ASW 1.7 software (Olympus, Japan). Representative images are shown.

In control cells, HER2 was homogeneously distributed throughout the cell. Combined treatments of 1-10 μg/ml Tz + 10^-6^M RA induced a granular distribution of the HER2 receptor, which was very pronounced in SKBR3 cells (Figure 8A) and less conspicuous in BT-474 cells (Figure 8B). Hence, the HER2 receptor maybe regrouping and internalizing for its subsequent degradation. The internalization was then confirmed by confocal microscopy (Figure 9). HER2 was located in the cell membrane and cytoplasm in control cells; in SKBR3 cells it was internalized and found perinuclearly after the simultaneous treatments (Figure 9). The internalization and subsequent degradation of the receptor could have a beneficial effect in deactivating these highly oncogenic pathways.

### Combined treatment with Tz and RA reduces expression of FAK and HER2 in SKBR3 and BT-474 cells

**Figure 10:**
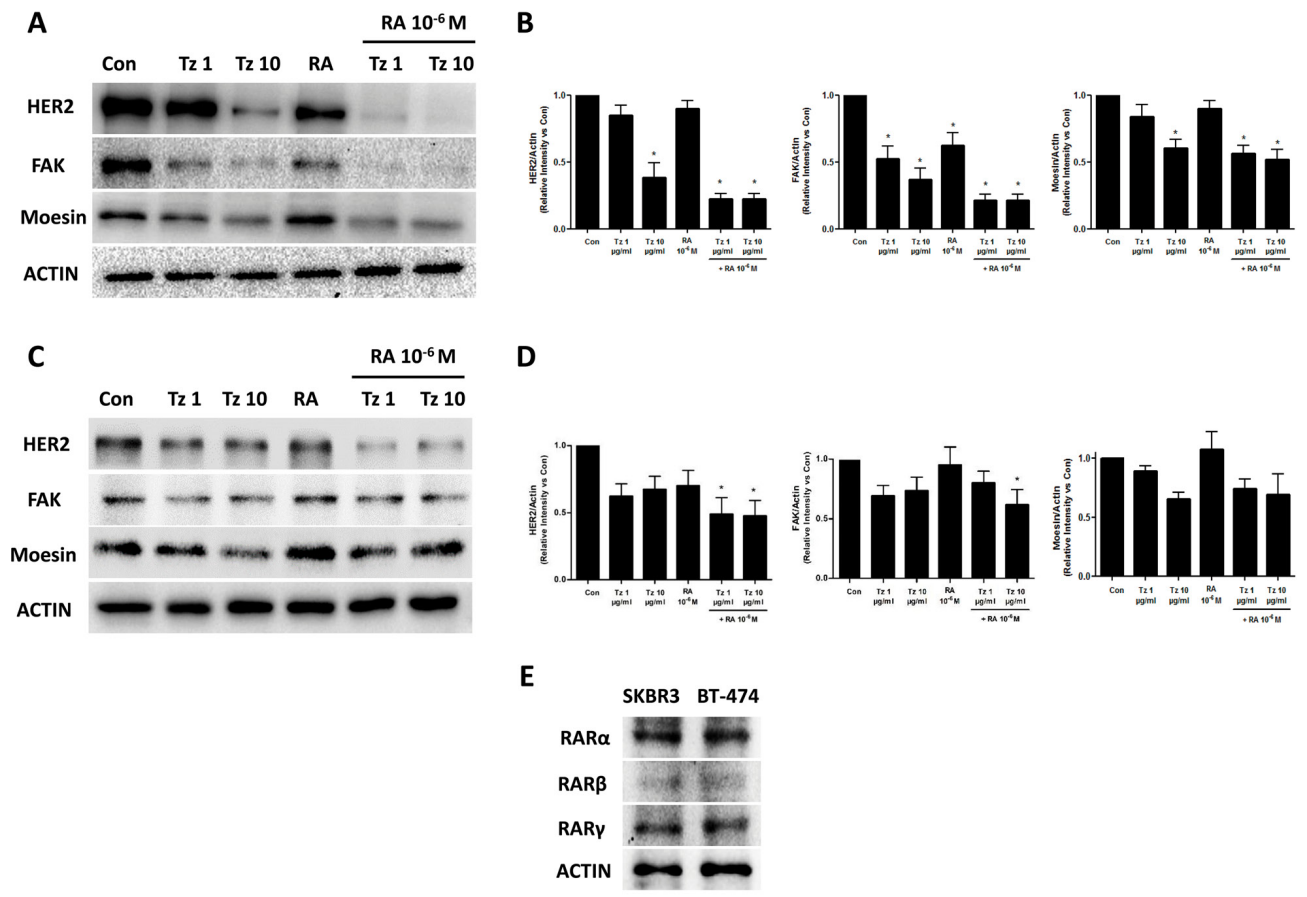
Tz and RA reduce HER2, FAK and Moesin expression **(A-B)** SKBR3 and **(C-D)** BT-474 cells were treated for 72 h with 1 or 10 μg/ml Tz, 10^-6^M RA or the combination of both drugs. Expression of HER2, FAK, Moesin and ACTIN was evaluated by Western blot. (B and D) HER2, FAK and Moesin densitometry values were adjusted to ACTIN intensity, respectively, then normalized to the control sample. ^*^= P<0.05 vs. corresponding control. **(E)** RARα, RARβ and RARγ expression were visualized in SKBR3 and BT-474 cells. ACTIN expression was used as charge control. Experiments were performed three times.

The expression of FAK, Moesin, and HER2 receptor was evaluated by Western blot after 72 h of treatment with 1-10 μg/ml Tz, 10^-6^M RA or the combination of both drugs. In SKBR3 cells, HER2 and Moesin expression significantly decreased following treatments with 10 μg/ml Tz and the combination of 1-10 μg/ml Tz with 10^-6^M RA. Expression of FAK was markedly reduced by all treatments and, to a lesser extent, by RA alone (Figure 10A–10B). In the BT-474 cell line, HER2 expression was reduced by all treatments although only the simultaneous treatments with 1-10 μg/ml Tz + 10^-6^M RA were statistically significant. The expression of FAK was significantly reduced by the combined application of 10 μg/ml Tz and 10^-6^M RA. Expression of Moesin was inhibited by treatments with 10 μg/ml Tz and the combination of both drugs, but these reductions were not statistically significant (Figure 10C–10D). Finally, we demonstrated a similar expression of RARα, RARβ and RARγ in SKBR3 and BT-474 cells (Figure 10E). Actin was used as load control and its expression was not modified with different treatments.

## DISCUSSION

In the last few decades, individualized treatment has played an increasing role in the management of breast cancer patients. Approximately 25% of invasive breast tumors have HER2 overexpression or amplification, which is an adverse prognostic factor [5]. Tz was approved by the Food and Drug Administration (FDA) for patients with advanced breast cancers that express HER2, and Tz is currently the therapy of choice and the most widespread treatment for this tumor subtype [7]. Cardiac dysfunction was, however, early recognized as a potential adverse consequence of Tz treatment. Furthermore, despite initial encouraging results, the response rate to Tz as a single agent in the first-line treatment of metastatic breast cancer revealed to be lower than 40%, and the median duration of response was between 9 and 12 months [6, 23]. This suggests that both de-novo and acquired resistance to Tz may occur.

Retinoids have been proposed as adjuvant treatment of breast carcinoma because they inhibit the growth of breast cancer cell lines and breast tumors in animal models. They also induce morphological or phenotypic differentiation in breast carcinoma cell lines [24, 25]. Combinations of retinoids with other drugs are of great interest given the disappointing results obtained with the use of the former as the only therapeutic agents in advanced disease [26]. The clinical and effective use of retinoids in patients with breast carcinoma requires the identification of subpopulations that would benefit from this therapy, as this type of tumor is highly heterogeneous and may lack the corresponding receptors. Preclinical and clinical data indicate that RARα levels determine the response to retinoids [27], and overexpression of RARα and HER2 has been reported in human breast tumors [15, 28].

Because retinoids and Tz are individual agents that have antitumor activity in breast cancer, combinations of these drugs may translate into improved therapy for patients who suffer this disease. Paroni et al. showed that in HER2+/RAR+ cells, the sensitivity to retinoids is further potentiated with the use of lapatinib, which targets HER1 and HER2 and inhibits their tyrosine kinase activity [15]. They suggested that a subgroup of patients with HER2+/RAR+ breast cancer could be treated with a new targeted anti-HER2 therapy combined with RA. These authors also proposed the combination of RA and lapatinib agonists as a promising therapy.

Our results demonstrate that the combination of RA and Tz exerts a strong synergism in the inhibition of cell viability in human HER2-overexpressing breast cancer cells SKBR3 (ER+/HER2+/RARα+) and BT-474 (ER-/HER2+/RARα+), suggesting that this combination could be beneficial for both ER-positive and negative tumors. Our results are in accordance with the findings of Koay et al. who reported the synergistic inhibition of cell growth in SKBR3 cells after combined treatment with Tz and RA [29] and with Tatebe et al. who demonstrated synergistic growth inhibition by 9-cis-retinoic acid plus Tz in human hepatocellular carcinoma cells [30]. These results suggest that the combined application of these two drugs might be useful in a wider range of malignancies. Our results also confirm Tari et al.’s observations that RA did not inhibit the growth of BT-474 cells, but combining RA with Tz enhanced the effect of Tz alone [31].

Koay et al. also analyzed the effect of RA and Tz on cell cycle in BT-474 and SKBR3 cells. They demonstrated that combinations of Tz with various retinoids lead to enhanced accumulation of BT-474 cells in the G0/G1 phase and of SKBR3 cells in the G2/M phase, coupled with a reduction in the S phase. The authors concluded that combinations generally lead to a greater reduction of cells in the S phase of the cell cycle than the respective single agents [29].

While the effects of Tz on cell proliferation, cell cycle, angiogenesis and apoptosis have been investigated in depth, its effect on cell migration has received little attention. Our study reveals that, although RA and Tz applied separately both decrease migration and invasion of SKBR3 and BT-474 cells, their combination exerts a higher inhibition on cell motility. Our group recently reported that RA inhibits the adhesion/migration of other breast cancer cell lines that do not overexpress HER2 [21, 22]. The inhibition of cell migration exerted by Tz was previously reported by Jeon et al. in SKBR3 and AU-565 cells, both HER2+. HER2 overexpression was shown to be correlated with increased fibronectin synthesis, which plays a central role in cell adhesion/migration. Treatment with Tz decreased fibronectin expression and led to decreased cell adhesion in SKBR3 cells, which was not observed in a HER2-negative cell line [32].

Another important finding of our work is that the combined treatment with Tz and RA induces a granular distribution of the HER2 receptor and its subsequent internalization, which was most evident in SKBR3 cells. Endocytosis has been recognized as the most significant pathway in the downregulation of human epidermal growth factor receptor family by removing the receptor from the cell surface for degradation in lysosomes. The predominant opinion is that Tz causes the endocytic downregulation of HER2 [4, 33]. Unlike other ErbB-2 family members that are internalized and degraded after stimulation, HER2 remains on the cell surface and continues to signal for prolonged periods [34]. This property is thought to contribute to HER2’s ability to transform cells when it is overexpressed. The coadministration of Tz and RA is more effective in inducing receptor clustering and internalization. This finding suggests that the receptor could be regrouping and internalizing for its subsequent degradation, which would have a beneficial effect by deactivating the cascade of this highly oncogenic pathway. Further studies with different endosomal-lysosomal markers are needed to confirm these results.

The major downstream signaling pathway activated by HER2 is PI3K/mTOR, and Src-FAK signaling is a potential integrator of receptor crosstalk. PI3K, Src and FAK have independently been implicated in Tz resistance [35]. Receptor crosstalk to HER2 (i.e., trans-phosphorylation of HER2 by other growth factor receptors) is an important mechanism by which HER2 can remain activated even in the presence of HER2-targeted therapy [35]. We explored FAK location by immunofluorescence and observed that Tz and RA induced nuclear FAK translocation in both cell lines. Focal adhesion complex formation in the cell membrane at specialized sites, such as ruffles and pseudopods, is essential for cellular migration, and FAK is key in this process. The nuclear localization of FAK reduced or inhibited the formation of such structures, suggesting that migration was eventually diminished. Our group has recently shown that RA treatment induces nuclear FAK translocation in T-47D and MCF7 cell lines, and we suggested that this translocation prevents correct cell motility [22]. In summary, the combined treatment would allow to attack the tumor cells with an anti-HER2 therapy but also block its intracellular activation by inducing FAK translocation to the nucleus, thus deactivating the signaling pathway at two different sites and eventually diminishing cellular migration.

Treatment with Tz also induced the rearrangement of vinculin, a cytoskeletal protein closely related with FAK that exhibits a similar subcellular distribution. Tz alters the staining pattern of vinculin, which is discrete and focal on the cell periphery in control cells, but disordered and clumpy both in the cytoplasm and at the periphery of cells treated with Tz [36]. These observations suggest that Tz could inhibit migration and disrupt the normal distribution of cytoskeletal proteins in breast cancer cells that overexpress HER2.

Finally, we examined the ability of Tz, RA and the combination of both to modify the expression of HER2, FAK and Moesin to elucidate the molecular mechanisms underlying the synergistic inhibition of growth, migration/invasion and protein relocalization. Tz and RA individually caused a reduction in the expression of FAK in SKBR3 cells. The combination of both drugs produced, however, a stronger inhibition of FAK and Moesin expression, which could contribute to the inhibition of cell migration. FAK is a central controller of cell migration, particularly in the setting of tumor metastasis. Overexpression of FAK is related to the metastatic behavior of various tumors, such as melanoma [37], lung [38] and ovarian cancer [39]. High FAK expression in human breast cancer is associated with an aggressive phenotype [40]. In a rat breast cancer metastasis model, inhibition of FAK activity abrogates cancer diffusion to the lung [41], and silencing of FAK in human and mouse mammary tumor cells results in loss of invasive ability [42]. Hence, a decrease in FAK expression/activity is somewhat beneficial or desired in the treatment of breast tumors. On the other hand, HER2 receptor expression was markedly lower after treatment with 10 μg/ml Tz or the combination of Tz with RA, and slightly lower with RA alone in SKBR3 cells; in BT-474 cells, HER2 expression was reduced by all treatments, but significantly only by the combination of both drugs. Our results evidence that the combination of Tz and RA is more effective in down-regulating the receptor expression than each drug alone because they engage distinct pathways. In accordance with our results, previous studies have shown that Tz reduces HER2 levels in addition to blocking its activation pathway [31], and that 10^-6^M RA decreases HER2 RNA levels and the expression of the protein on the cell surface by 20-30% in SKBR3 cells [43].

The use of multiple drugs may target several objectives or subpopulations simultaneously. The potential favorable outcomes for synergism include 1) increasing the efficacy of the therapeutic effect, 2) reducing the dosage but increasing or maintaining the same efficacy to avoid toxicity, and 3) minimizing or slowing down the development of drug resistance. Drug combinations have been widely used to reach these therapeutic benefits and have become the leading choice for treating the most dreadful diseases, such as cancer [44]. The combination of Tz and RA is promising for at least two reasons: first, because of the highly synergistic interactions that occur through the combined action of both drugs. Second, because the combined treatment would be less toxic because the Tz doses applied would be lower due to the adjuvant effect of RA. The combination of RA with Tz induces a strong and synergistic inhibition of cell survival, accompanied by a decrease in adhesion/migration/invasion in BT-474 and SKBR3 cells. The Tz and RA combination resulted in a potent inhibition of the expression of FAK and a down-regulation of HER2 receptor.

In conclusion, treatment with RA and simultaneous inhibition of HER2 signaling may be a promising therapy for patients with HER2 positive breast cancer. Our findings suggest that the coadministration of both drugs in patients with this type of cancer could contribute to improving their prognosis and reduce the adverse effects of the conventional therapy. It may also reveal new strategies to overcome endocrine therapy resistance. Our work provides new approaches that justify the design of new clinical trials to evaluate the combination of anti-HER2 and RA therapies in patients with HER2+/RAR+ breast cancer. It also provides new insights into the biology of this tumor subtype, defining novel molecular determinants and pathways involved in neoplastic cell survival/adhesion and migration.

## MATERIALS AND METHODS

### Cell cultures and treatments

The human breast carcinoma cell lines SKBR3 (ER-/HER2+/RARα+) and BT-474 (ER+/HER2+/RARα+) were obtained from the American Type Culture Collection (Rockville, MD, USA). SKBR3 and BT-474 cells were routinely grown in RPMI 1640 supplemented with L-glutamine (2 mM) and 10% fetal bovine serum (FBS, Gibco^®^) at 37°C and 5% CO_2_. Humanized monoclonal antibodies Tz (Herceptin, Roche) stock solution was prepared in sterilized apyrogenic water at a concentration of 1000 μg/ml, preserved at 4°C for short-term or at -20°C for long-term storage and protected from light. Tz was used in a final concentration of 1 μg/ml and 10 μg/ml, dissolved in RPMI 1640 culture medium. All-*trans*-retinoic acid (RA) was obtained from Sigma-Aldrich (St. Louis, MO, USA). RA stock solution was dissolved in dimethyl sulfoxide (DMSO) at a concentration of 10^-2^M and maintained at -20°C, protected from light and in an inert atmosphere. It was used at a final concentration of 10^-6^M, according to our previously published results [21], and prepared in RPMI 1640 culture medium. All experiments with retinoids were performed in light-reduced room.

### Viability assay

The MTT [3-(4, 5-dimethylthiazol-2-yl)-2,5-difeniltetrazol] (Sigma-Aldrich, St. Louis, MO, USA) was dissolved in RPMI 1640 culture medium at a concentration of 5 mg/ml. The working solution was 0.5 mg/ml MTT. SKBR3 and BT-474 cell lines were seeded into 96 well plates at a density of 20,000 cells/well and 32,000 cells/well, respectively. Treatment with 0.1-100 μg/ml Tz, 10^-8^-10^-4^M RA or the combination of both drugs was performed. After 24-72 h, the medium was removed and the cells were incubated with 100 μl MTT/well for 4 h. MTT was removed and the formazan crystal rings were dissolved in 100 μl DMSO. Absorbance at 570 nm was measured by using a microplate reader (MULTISKAN EX; Thermo Scientific, Lafayette, CO, USA).

### Pharmacological interaction analysis

We used the CompuSyn software to characterize the pharmacological interaction produced by treatment with 1-10-100 μg/ml Tz in combination with 10^-6^M RA. This program utilizes the combination index (CI) as a method to quantify cytotoxic drug synergism based on the mass-action law designed by Chou and Talalay [44, 45]. Synergism and antagonism were defined as a more or a less than expected additive effect, respectively. CI, affected fraction (Fa), and dose-reduction index (DRI) levels were calculated from the effects of varying doses on cell viability inhibition rates in the MTT assay. CI =1 denotes an additive effect, CI <1 synergism, and CI >1 antagonism. The interaction of different Tz+RA combinations in both cell lines was expressed as isobolograms. The affected fraction (Fa) is obtained by the following equation: 1 - (% survival or unaffected fraction / 100%), with 1 corresponding to 100% cell survival. DRI is a measure of how much the dose of each drug, in a synergistic combination, can be reduced to obtain a given biological effect when compared with the doses for each drug alone. Although a DRI >1 is beneficial, it does not necessarily indicate synergism; it is, however, important from a clinical stand point where dose reduction predicts reduced toxicity toward the host while retaining therapeutic efficacy.

### Cell adhesion assay

Cells were exposed to 1-10 μg/ml Tz, 10^-6^M RA or the combination of both drugs for 72 h. Then, 10,000 SKBR3 cells/well or 50,000 BT-474 cells/well were seeded into 96-well plates previously coated with 1% sterile gelatin (Sigma-Aldrich). The cells were incubated at 37°C in a tissue culture incubator. The culture medium was removed after 1 h and washed with PBS to remove any non-adherent cells. The attached cells were fixed/stained with 10% ethanol/crystal violet for 20 min. Absorbance at 570 nm was measured with a microplate reader (MULTISKAN EX; Thermo Scientific, Lafayette, CO, USA) and images of attached cells were captured by a Nikon Eclipse E200 microscope coupled to a high-resolution 590CU 5.0M CCD digital camera.

### Cell migration assay (wound healing assay)

A scratch wound assay was conducted to assess the influence of Tz and RA on cell migration. SKBR3 and BT-474 cells were seeded in 96-well plates and incubated until 80% confluence. Wounds were made in the monolayers by scratching the surface with a pipette tip (10 μl) as uniformly and straight as possible. The cells were washed and the treatments prepared in 200 μl of medium added. Cell migration was monitored for 72 h. Digital images from cells were taken with a 16X objective after 24, 48 and 72 h. The distance of migration was then analyzed by phase-contrast microscopy and closed areas were quantified using imageJ software.

### Cell invasion assay

Cell invasion was assayed using the BD BioCoat™ Growth Factor Reduced (GFR) Matrigel™ Invasion Chamber (BD Bioscience, USA). Cells were exposed to 1-10 μg/ml Tz, 10^-6^M RA or the combination of both drugs for 72 h. Then, after rehydrating the GFR Matrigel inserts, 0.5 ml of cell suspension (15,000 SKBR3 cells or 300,000 BT-474 cells) was added to the inside of the inserts. The chambers were incubated for 72 h at 37°C, 5% CO_2_ atmosphere. After incubation, the non-invading cells were removed from the upper surface of the membrane using cotton-tipped swabs. The cells on the lower surface of the membrane were then fixed/stained with 10% ethanol/crystal violet for 20 min. The invading cells were observed, photographed under the microscope at 100X magnification and counted in the central field.

### Transfection experiments

The synthetic small interfering RNA for FAK (siRNA SMART pool FAK) was used. SKBR3 and BT-474 cells (70-80% confluent) were transfected with 50–75 nM of target siRNA by using Lipofectamine (Invitrogen, Carlsbad, CA, USA) together with the treatments. Two pulses were performed: one of 24 h, after which a new pulse (50% of the initial pulse) was added for 72 h to maintain the silencing. The FAK inhibitor (FAKi, sc203950A, Santa Cruz) was used in a final concentration of 1 μM in combination with the treatments.

### Immunofluorescence

SKBR3 and BT-474 cells were grown on coverslips previously coated with 1% sterile gelatin (Sigma-Aldrich) and exposed for 72 h to 1-10 μg/ml Tz, 10^-6^M RA or the combination of both drugs. Cells were fixed with 4% paraformaldehyde for 35 min and permeabilized with 0.1% Triton for 5 min. Blocking step was performed with 0.5% bovine serum albumin solution for 30 min at room temperature. Cells were incubated with the first antibody against FAK (Mouse, clone 77, BD Biosciences) and against HER2 (Mouse, catalogue 16901-Abcam) overnight at 4°C. After washing, cells were incubated with Goat Anti-Mouse IgG-Alexa Fluor 488 (A-11001, Invitrogen) for 90 min at room temperature. The cells were washed and then stained for 35 min with Texas Red phalloidin (Sigma-Aldrich) to reveal actin and the nuclei counter stained with 40-6-diamidino-2-phenylindole (DAPI, Sigma-Aldrich) for 10 min. The coverslip cells were mounted with Vectashield mounting media (Vector Laboratories, Burlingame, CA, USA). Immunofluorescence images were captured by using a Nikon Eclipse E200 microscope (Tokyo, Japan) coupled to a high-resolution 590CU 5.0M CCD digital camera or examined under fluorescence microscopy (FV1000 Olympus Confocal Microscope) and the FV 10-ASW 1.7 software (Olympus, Japan).

### Immunoblotting

Cell lysates were separated by SDS-PAGE. Antibodies used were: Mouse Anti-FAK (610088, BD Biosciences), Goat Anti-Moesin (C-15 sc-6410), Goat Anti-ACTIN (C-11), Mouse Anti-RARα (C-1 sc-515796), Rabbit Anti-RARβ (C-19 sc-552), Mouse Anti-RARγ (G-1 sc-7387) (Santa CruzBiotechnology) and Mouse Anti-HER2 ab16901 (Abcam). Primary and secondary antibodies were incubated with the membranes by means of standard techniques. Immunodetection was accomplished with enhanced chemiluminescence. The images were captured by using ChemiDoc TM XRS+ System with Image LabTM Software #170-8265 (Bio-Rad, Hercules, CA, USA).

### Statistical analysis

Statistical analysis of the data was performed with one-way ANOVA followed by Tukey-Kramer Multiple-Comparisons test (GraphPad PRISM software version 5.03, San Diego, CA, USA). P <0.05 was considered statistically significant. All values were expressed as mean ± SD.

## SUPPLEMENTARY MATERIALS FIGURES



## Abbreviations

(Tz): Trastuzumab
(RA): All-*trans*-retinoic acid
MTT: [3-(4, 5-dimethylthiazol-2-yl)-2,5-difeniltetrazol]
(HER2): human epidermal growth factor receptor 2
(ErbB-2, also known as HER2): human epidermal growth factor receptor 2
(RAR): retinoic acid receptors
(RXRs): retinoid X receptors
(ER): estrogen receptor
(FAK): focal adhesion kinase
(FAKi): FAK inhibitor
(Fa): fraction affected
(CI): combination index
(DRI): dose reduction index
(PI3K/mTOR): Phosphatidylinositol-4,5-bisphosphate 3-kinase/mammalian Target of Rapamycin
(FBS): fetal bovine serum
(DMSO): dimethyl sulfoxide
