# Correction: Synergistic antitumor activity by combining trastuzumab with retinoic acid in HER2 positive human breast cancer cells

**DOI:** 10.18632/oncotarget.28345

**Published:** 2023-01-26

**Authors:** Fiorella Vanderhoeven, Analía Lourdes Redondo, Ana Laura Martinez, Laura María Vargas-Roig, Angel Matias Sanchez, Marina Inés Flamini

**Affiliations:** ^1^Instituto de Medicina y Biología Experimental de Cuyo, Centro Científico Tecnológico, Mendoza, Argentina; ^2^Facultad de Ciencias Médicas, Universidad Nacional de Cuyo, Mendoza, Argentina

**This article has been corrected:** In Figure 4A, the top row, 3rd panel image is an accidental duplicate of the bottom row, 3rd panel image in Figure 4C. The corrected Figure 4, obtained using the original data, is shown below. The authors declare that these corrections do not change the results or conclusions of this paper.

Original article: Oncotarget. 2018; 9:26527–26542. 26527-26542. https://doi.org/10.18632/oncotarget.25480

**Figure 4:**
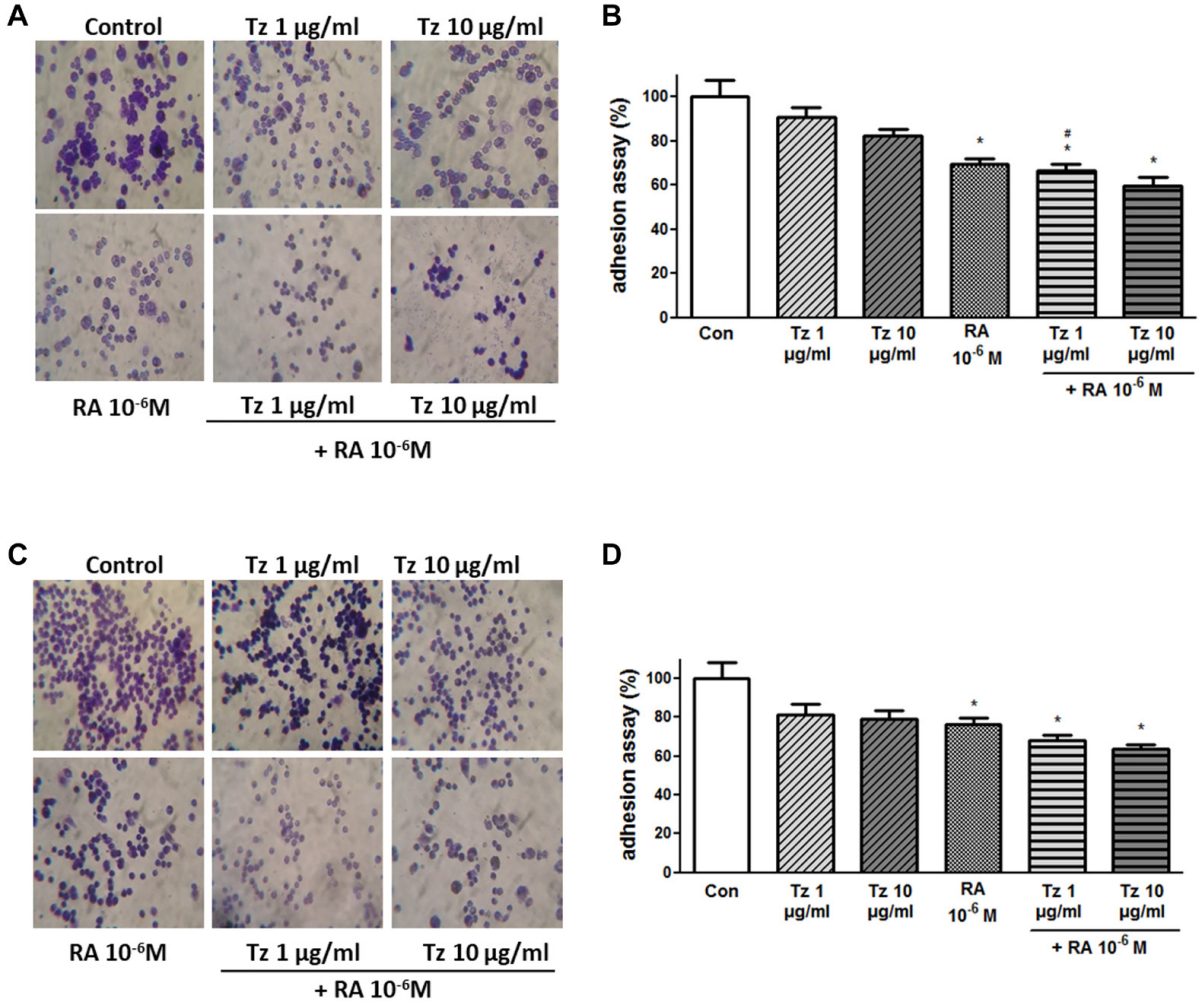
RA and the combination of both drugs inhibit cell adhesion. (**A**, **B**) SKBR3 and (**C**, **D**) BT-474 cells were treated for 72 h with 1–10 μg/ml Tz, 10^−6^M RA or the combination of both drugs. After the treatment, cells were placed on coverslips previously covered with gelatin and a cell adhesion assay was performed. (A, C) Representative images of the adhered cells. (B, D) Percentage of attached cells (absorbance at 570 nm). Experiments were performed in triplicate. ^*^ *P* < 0.05 vs. Control (Con). ^#^ *P* < 0.05 vs. 1 μg/ml Tz.

