# Supplementary material for: Synergistic antitumor activity by combining trastuzumab with retinoic acid in HER2 positive human breast cancer cells
Source: Oncotarget. 2018 May 29;9(41):26527–42. doi: 10.18632/oncotarget.25480 (PMC5995169; doi:10.18632/oncotarget.25480)
Supplement: Supplementary file 1 [file oncotarget-09-26527-s001.pdf]

# Synergistic antitumor activity by combining trastuzumab with retinoic acid in HER2 positive human breast cancer cells

## SUPPLEMENTARY MATERIALS

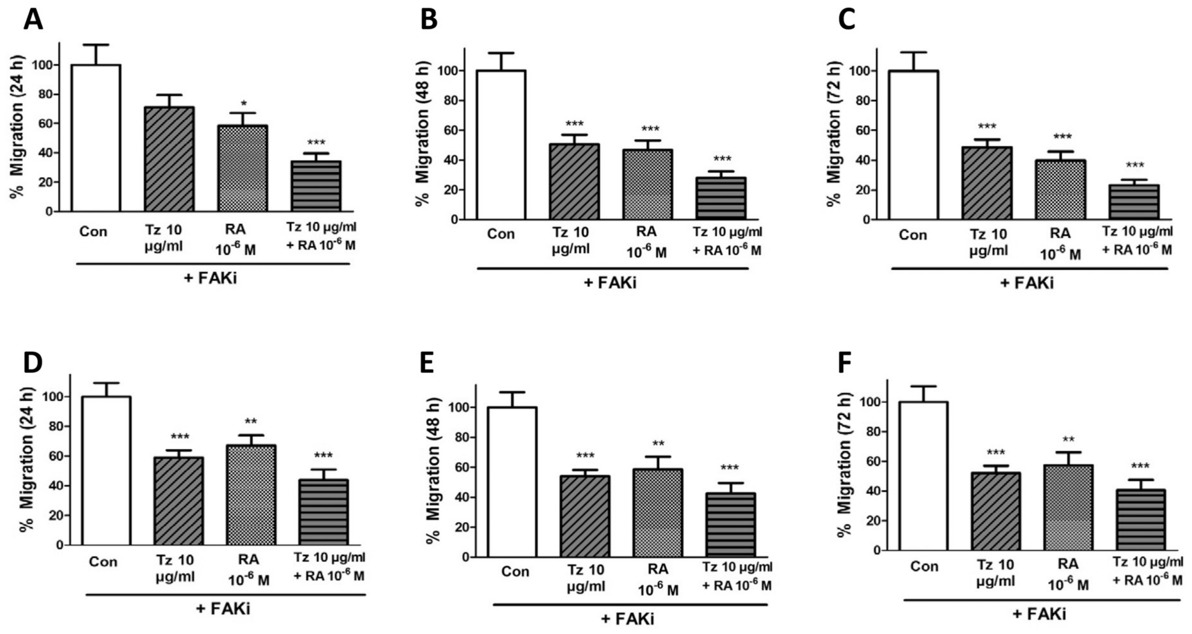


**Supplementary Figure 1: Tz and RA also reduce cell migration in the presence of FAK inhibitor.** **(A-C)** SKBR3 and **(D-F)** BT-474 cells were grown in 96-well plates until they formed a monolayer; then, a "wound" was made. The treatments, in the presence of FAK inhibitor (FAKi), were performed for 72 h; cell migration was monitored by taking photographs. (A, D) 24 h (B, E) 48 h (C, F) 72 h of treatment. Gap closure was quantified with the use of NIH image J software. Experiments were performed in triplicate. $^{*}P < 0.05$ vs. Control (Con).

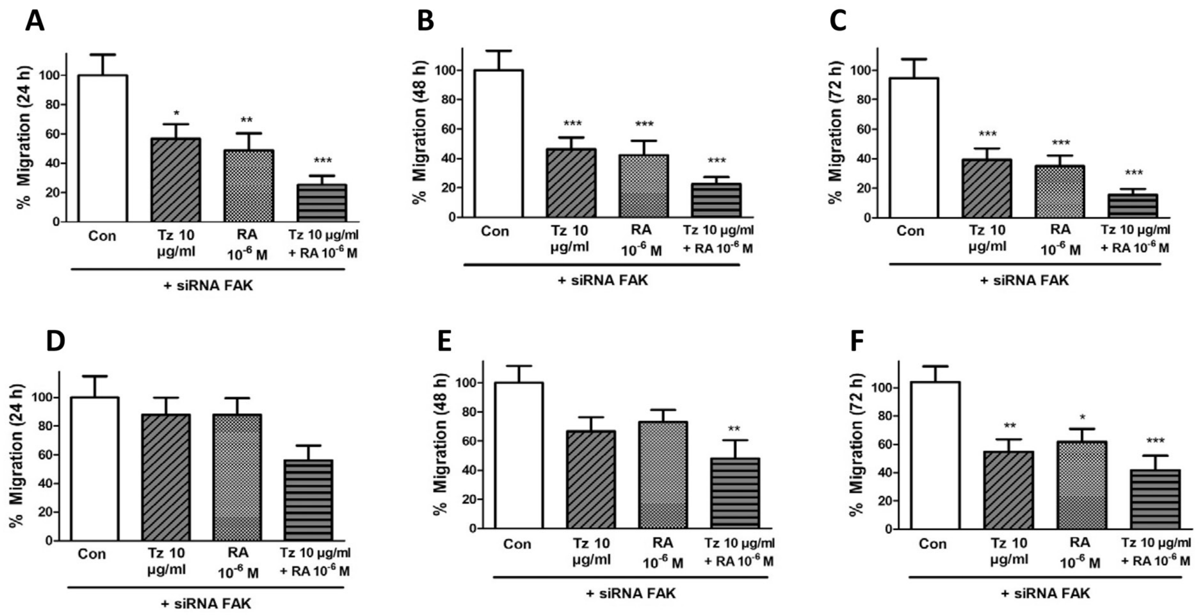


**Supplementary Figure 2: Tz and RA also reduce cell migration in the presence of specific FAK siRNA. (A-C)** SKBR3 and **(D-F)** BT-474 cells were grown in 96-well plates until they formed a monolayer; then, a "wound" was made. The treatments, in the presence of specific FAK siRNA, were performed for 72 h; cell migration was monitored by taking photographs. (A, D) 24 h (B, E) 48 h (C, F) 72 h of treatment. Gap closure was quantified with the use of NIH image J software. Experiments were performed in triplicate. $^{*}P < 0.05$ vs. Control (Con).
